# Three-Point Bending Properties of Hybrid Multi-Materials Using Adhesive Bonding Dependent on Strength Difference between Steel and Aluminum

**DOI:** 10.3390/ma15093328

**Published:** 2022-05-06

**Authors:** Geonwoo Jeon, Dongwoog Ha, Yunmin Park, Changyeol Jeong

**Affiliations:** Department of Nuclear and Energy System Engineering, Dongguk University, Dongdae-ro 123, Gyeongju-si 38066, Gyeongsangbuk-do, Korea; wjsrjsdn9@gmail.com (G.J.); gkehddnr8612@naver.com (D.H.); ssoowa12@naver.com (Y.P.)

**Keywords:** steel, aluminum, multi-materials, adhesion, mechanical property, finite-element analysis

## Abstract

The mechanical behaviors of two multi-materials, DP590 (steel sheet)–A356 (cast aluminum alloy) and SS330 (steel sheet)–A5052 (aluminum sheet), were studied. A structural adhesive was used for the joining of steel and aluminum at adhesion strengths of 10, 22, and 30 MPa. To demonstrate that the three-point bending properties depend on the difference in strength between steel and aluminum and adhesion strength, optical microscopy (OM), scanning electron microscopy (SEM), and finite-element analysis (FEA) were performed. According to the results of the bending tests on both multi-materials under the same stacking conditions, the flexural stress increased with the improvement in the adhesion strength until interface separation or aluminum fracture. At the same adhesion strength, the DP590 (lower)–A356 (upper) and SS330 (upper)–A5052 (lower) configurations exhibited a tendency to decrease in the sudden stress drop due to aluminum fracture and interface separation. The bending results were analyzed through the FEA and the stress distribution as a function of the stacking and adhesion strength was confirmed.

## 1. Introduction

Higher vehicle efficiencies are required to improve the fuel economy and reduce emissions worldwide. In addition, the automobile industry has been confronted with strengthened vehicle safety regulations [1,2]. The combination of two different materials to satisfy the demands has attracted attention worldwide. Various joining methods for hybrid multi-materials have been employed, such as welding [3,4], cladding [5,6], riveting [7,8], and adhesive bonding [9,10,11,12]. The melting points of the two materials are different in the commonly used welding, which causes material property loss [13,14]. The increase in rivet strength is limited because rivets require an adequate ductility and high strength [15]. In addition, the method of mechanical fastening should consider the plasticity of the two materials and is hindered by challenges such as stress concentration and the fastening part cracking due to fastening pressure [16]. Structural adhesives have been used for joining to compensate for the disadvantages of these joining techniques. Compared to other joining methods, the adhesive bonding method has the following advantages [17,18,19]. First, the stress is evenly distributed throughout the adhesion surface to prevent stress concentration. Second, chemical corrosion between steel and aluminum can be prevented. Third, the adhesive bonding has an excellent fatigue resistance compared to those of riveting or welding and excellent reductions in noise. Among the numerous types of adhesives, structural adhesives are generally selected owing to their high strengths.

In the automotive industry, adhesives are already applied to structural units [20,21]. Studies on the improvements in adhesive strength and mechanical properties are being performed to enhance crash properties. Extensive studies have been carried out to improve the adhesion strength [9,10,11,12,22,23,24]. Several studies have been carried out on the mechanical properties of multi-materials [25,26,27]. Alfano et al. [25] used two types of epoxy adhesives and evaluated the fracture toughness of T-joints using different thicknesses of a cold-rolled galvanized steel (FeP04). They reported that the adhesive thickness and steel thickness had no significant effects on the adhesive fracture toughness. Azari et al. [26] studied the effects of the mode ratio of load, adhesion thickness, substrate modulus, ejection fillet, and surface roughness on the fatigue threshold and crack growth rate. They reported that the fatigue crack initiation was insensitive to the detailed shape of the adhesive fillet. The effect of the adhesive thickness on the fatigue behavior became more pronounced as the applied strain energy release rate and crack growth rate increased. Loureiro et al. [27] studied the stiffness, strength, impact, damping, and fatigue life based on different types of adhesives, such as elastic, polyurethane, and epoxy, through single-lap joint and T-joint tests. They reported that the epoxy adhesive has higher stiffness than the polyurethane in the single lap joint test. Also, the stiffness of the T-joint with the polyurethane is much lower than that with the epoxy. Maurel-Pantel et al. [28] evaluated the effect of adhesive thickness on mechanical properties. Also, a parametric analysis was performed to define a new formula able to describe the nonlinear response of the bonded connection in terms of vertical deflection at varying adhesive properties. However, extensive studies on the bending properties as a function of the stacking conditions and adhesion strength of multi-materials joined with adhesives are lacking. Therefore, studies on the bending properties and fracture behaviors of multi-materials are required to actively utilize joining using adhesives in the automotive industry.

The aim of this study was to compare the mechanical properties of two types of multi-materials joined with a structural adhesive. If only high-tensile steel is used, there is a weight increase and energy absorption properties are deteriorated. Therefore, the use of multiple materials and bonding methods are important. Bending properties are an important standard for evaluating the resistance to impacts and fractures applied to transport and vehicles. In addition, it is necessary to study the characteristics of the bonding of the multi-materials related to the components of renewable energy systems, such as wind power. For example, the blades of a wind turbine are bent due to the wind, and thus the bending characteristics must be evaluated. A DP590 steel plate and A356 aluminum casting alloy used as chassis parts were joined with a structural adhesive. DP590 is widely used as a structural material for automobiles by combining high tensile strength and excellent formability. In addition, A356 aluminum casting alloy is widely used in the automotive industry due to its excellent corrosion resistance and mechanical properties. Three-point bending tests were then carried out according to the adhesion strength. To compare the bending properties dependent on the difference in strength between steel and aluminum, SS330 steel plate and A5052 aluminum plates were joined and analyzed in terms of tensile and bending properties. In addition, the fracture behaviors of the multi-materials were analyzed by optical microscopy (OM) and scanning electron microscopy (SEM). Furthermore, a finite-element analysis (FEA) was performed to predict the distribution of stress and bending properties depending on the adhesion strength and stacking conditions. The aluminum fracture and interface separation were analyzed depending on the displacement in the three-point bending test.

## 2. Materials and Methods

### 2.1. Materials and Microstructures

Two types of steel, DP590 and SS330, and aluminum, A356 and A5052, were used. Table 1 lists the chemical compositions of DP590 (ASTM A1088 standard), SS330 (ASTM A36 standard), A356 (ASTM B26 standard), and A5052 (ASTM B209 standard). The three-point bending test specimens were processed to a thickness of 1.8 mm for each DP590, SS330, A356, and A5052 sheet.

**Figure 1:**
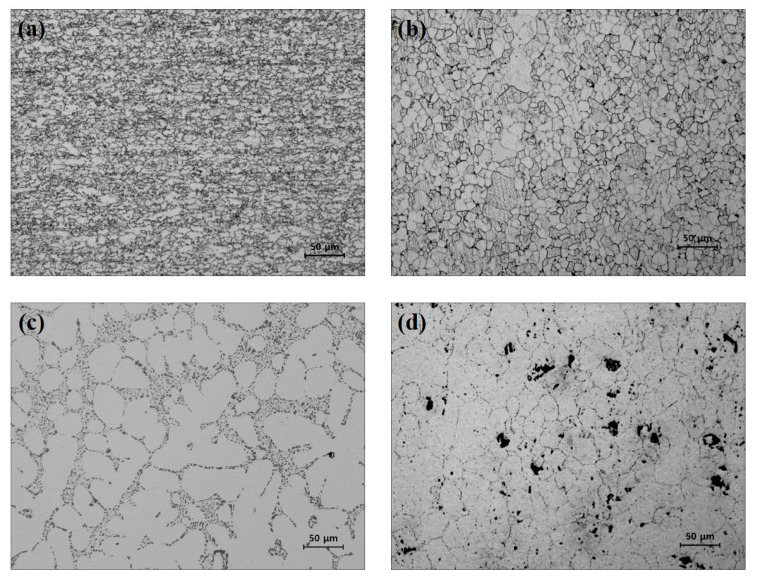
OM images of the microstructures of (**a**) DP590, (**b**) SS330, (**c**) A356, and (**d**) A5052.

To understand the characteristics of the base metal, the microstructures of DP590, SS330, A356, and A5052 were analyzed using OM (Nikon MA100, CFI60/CFI60-2 system). As shown in Figure 1a, the DP590 steel sheet microstructure consists of martensite mixed with a fine ferrite matrix and has an average grain size of approximately 9 μm. SS330 is a general structural steel, as shown in Figure 1b. The matrix consists of ferrite and pearlite with an average grain size of 15 μm. Figure 1c shows the microstructure of the as-received A356 cast alloy. The A356 alloy is generally composed of an α-aluminum dendrite phase, eutectic Si, precipitates, and Fe-based intermetallic compounds. The dendritic arm spacing of the A356 alloy used in this study was approximately 34 μm. Figure 1d shows the microstructure of A5052, which is an Al–Mg aluminum alloy with a grain size of 53.9 μm. In addition, the white part is an Al matrix, the gray part is Fe-rich particles, and the black part is a Mg_2_Si matrix.

### 2.2. Adhesion Strength and Surface Roughness

The structural adhesives used in this study were 2216 B/A Gray and DP460 epoxy adhesives (3M Science). The adhesion strengths of the two adhesives were evaluated using the American Society for Testing and Materials (ASTM) D1002 standard [29]. This test method covers measurement of the apparent shear strength of adhesives for metal bonding when tested under standard single lap joint specimens and specified preparation and test conditions. The material used to measure the adhesion strength was a steel–aluminum multi-material (DP590–A356 or SS330–A5052). The adhesion strength test was performed more than 10 times for each condition and yielded values of 10, 22, and 30 MPa. For the case of 10 MPa, the joint surface was uniformly polished in the length direction using a SiC paper #2000 (FEPA P-grade 2000) and cured at 66 °C for 2 h using a 2216 B/A Gray adhesive. In the case of 22 MPa, it was cured at 71 °C for 1.5 h using the DP460 adhesive after polishing using a SiC paper #2000. In the case of 30 MPa, the adhesion surface was ground in a direction perpendicular to the length using a mill file and cured at 81 °C for 8 h using the DP460 adhesive.

Two types of surface treatments, mill file and SiC paper #2000 treatments, were used for the specimen. The surface treatments of DP590 and A356 were also performed using a mill file and SiC paper #2000. The average arithmetic height, denoted as *Ra*, was measured using a surface roughness measuring instrument (Mahr Metrology Perthometer PGK 120, residual Rz values < 30 nm at a traversing speed of 0.1 mm/s). The roughness was measured five times per specimen. The measurement standard was International Organization for Standardization (ISO) 4287 [30].

### 2.3. Three-Point Bending Test

**Figure 2:**
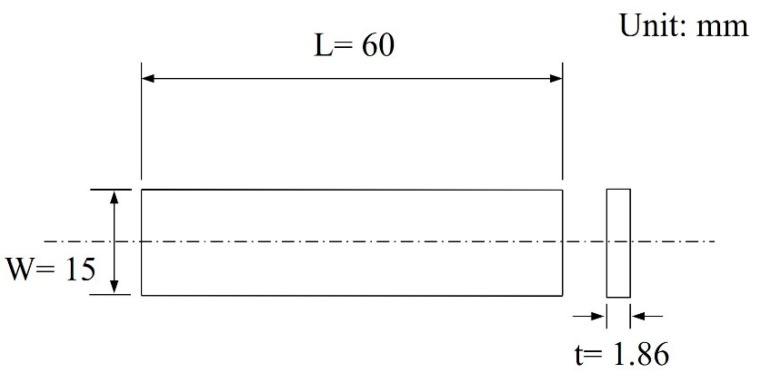
Shape of the bending test specimen (ASTM E290) [31].

**Figure 3:**
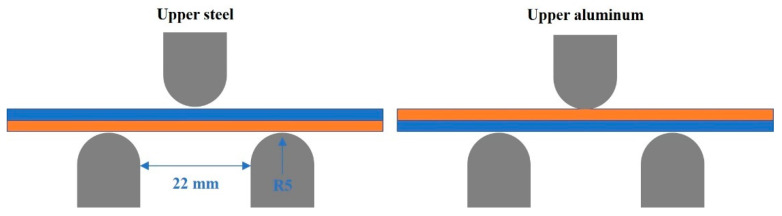
Structure of the bending test.

Figure 2 shows the shape of the bending test specimen according to ASTM E290 [31]. Bending specimens were manufactured into plate shapes with a width of 15 mm and a length of 60 mm. The number of tests was two to three for reliability. The bending test was carried out at a displacement speed of 5 mm/min and stopped at a displacement of 7.5 mm. Figure 3 shows a schematic of the three-point bending test. After the bending tests, the fracture behaviors were analyzed using a stereomicroscope (KEYENCE VHX-1000, real-time image stitching up to 10,000 × 10,000 pixels) and SEM (JEOL JSM-IT100, 5×–300,000× magnifications).

### 2.4. FEA

The bending test were simulated by FEA using ABAQUS to confirm the stress distribution according to the stacking configuration and adhesion strength. To verify the validity of the analysis, the results of the bending test and FEA were compared.

**Figure 4:**
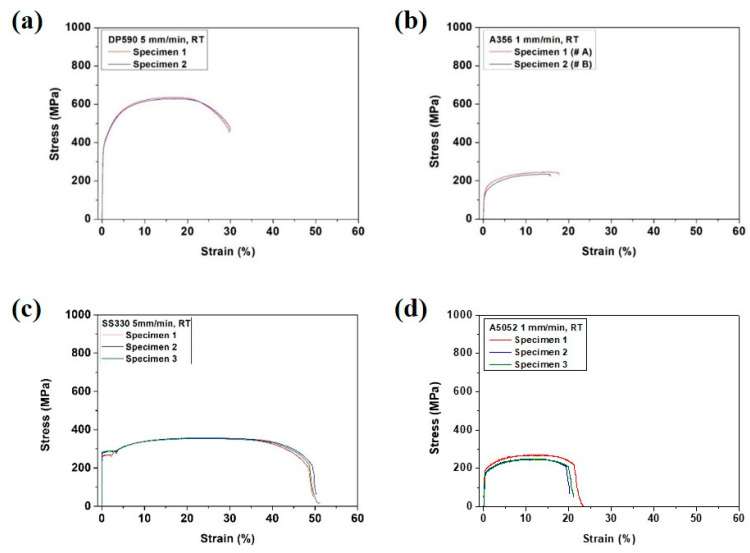
Stress–strain curves of the single (**a**) DP590, (**b**) A356, (**c**) SS330, and (**d**) A5052.

The FEA was carried out using two-dimensional (2D) shell modeling to reduce the analysis time. A CPS4R (four-node bilinear plane stress quadrilateral) element was used to set the plane strain conditions. The number of nodes was 7026 and the number of elements was 3804 for each material. The mechanical properties of each material used in this study are shown in Figure 4. The Poisson ratio for all materials was 0.3. Figure 4 shows the stress–strain curves of each single material. The yield strength (YS), ultimate tensile strength (UTS), and elongation (EL) of each single material are listed in Table 2. The difference in UTS between DP590 and A356 was approximately 400 MPa, while that between SS330 and A5052 was 100 MPa. A356, which has a low ductility, fractured immediately without necking. A5052, which has a higher ductility than that of A356, fractured with extension after necking.

The bending pin was modeled as an analytic ridge. Hard contact was set for normal behavior at the interface between the pin and the multi-material to assign the zero-penetration condition. The contact between the interface of the steel and aluminum exhibited cohesive behaviors at 10 and 30 MPa. Also, to define the cohesive behavior of adhesive strengths of 10 and 30 MPa, the traction-separation behavior was assumed as the stiffness coefficients for each adhesive strength. The cohesive behavior values corresponding to adhesive strengths of 10 and 30 MPa were used by deriving similar values from the experimental results. In addition, the extended finite-element method was applied to determine the failure behavior of A356 where the fracture occurred. Maximum principal stress (MAXPS) and maximum principal strain (MAXPE) were used for the damage modeling of A356.

## 3. Results and Discussion

### 3.1. Surface Roughness

**Figure 5:**
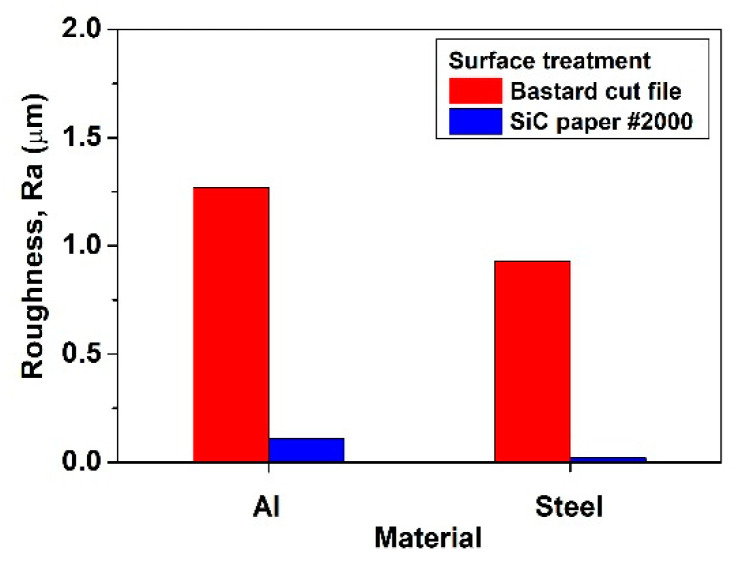
Surface roughnesses of the steel and aluminum.

As a result of the adhesion strength of each of the multi-materials, the average value of the adhesion strength was measured equally. To improve the adhesion strength from 22 to 30 MPa, the surface roughness of steel and aluminum, the curing temperature and time were increased. This indicated that the adhesion area was widened as the surface roughness increased [32]. The adhesion strength is affected by humidity, and the curing temperature and time were increased to decrease the humidity. This effect leads to an increase in adhesive strength. The surface roughness effect of the adhesion area for steel and aluminum is presented as a parameter, Ra, in Figure 5 and Table 3. In the case of surface treatment with a bastard cut file, the Ra value was 0.93 μm for steel and 1.27 μm for aluminum. When the surface was polished with SiC paper #2000, steel was 0.02 μm and aluminum was 0.11 μm. These results were similar to those in other literature [33,34,35]. When the surface was polished with SiC paper #2000, the roughness of cold working mold tool steel SKD11 was 0.023 μm [33] and the roughness of an Al-Cu-Mg alloy was 0.277 μm [34]. Hussein Zein et al. [35] stated that the surface roughness value decreased as the hardness increased. Our experiment also showed similar results, as aluminum has a higher roughness value than steel. Variables for improving the adhesion strength include the surface roughness, types of adhesives, curing time, and temperature and humidity, and systematic research is needed depending on these variables. Also, it is necessary to study the bending properties which allow for more diverse adhesive strength and the type of base material.

### 3.2. Bending Properties

**Figure 6:**
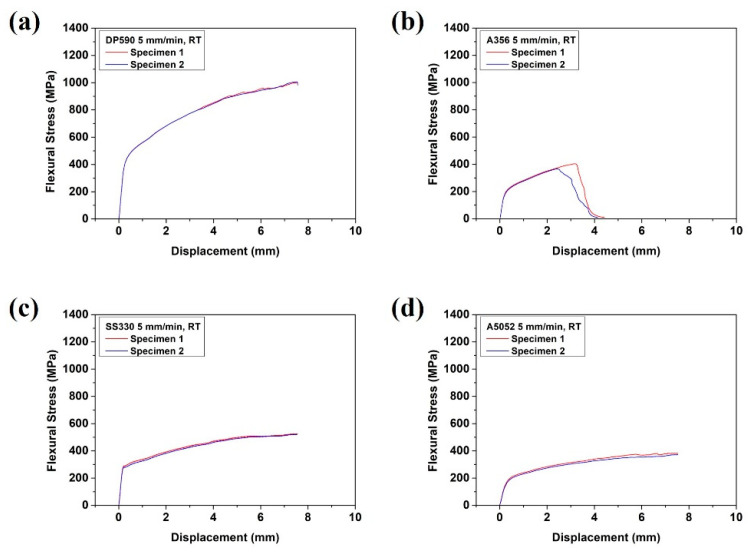
Single-material flexural stress–displacement curves for (**a**) DP590, (**b**) A356, (**c**) SS330, and (**d**) A5052.

Figure 6 and Table 4 show the results of the bending test on a single material prior to the bending test on the multi-materials. In Figure 6, the flexure stress–displacement curve of each single material shows that DP590, SS330, and A5052 were deformed without fracture until the end of the test, whereas A356 was fractured at a displacement of 2 to 4 mm.

**Figure 7:**
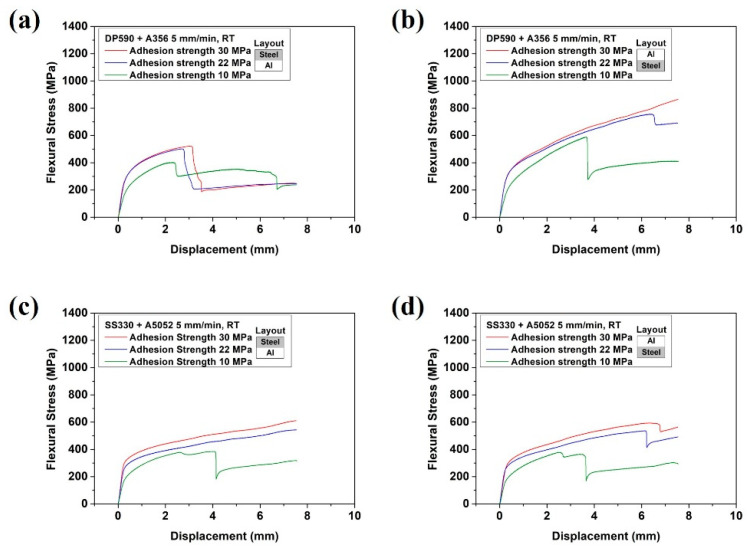
Flexural stress–displacement curves of both multi-materials with different adhesion strengths: (**a**) DP590 (upper)–A356 (lower), (**b**) DP590 (lower)–A356 (upper), (**c**) SS330 (upper)–A5052 (lower), and (**d**) SS330 (lower)–A5052 (upper).

Two stacking structures were used for the bending test. The first was steel (upper)–aluminum (lower), in which the steel was compressed, while the aluminum was tensioned. The second was steel (lower)–aluminum (upper), in which the steel was tensioned, while the aluminum was compressed. Figure 7a shows the bending results of the DP590–A356 multi-materials with different adhesion strengths in the steel (upper)–aluminum (lower) configuration, while Figure 7b shows those in the steel (lower)–aluminum (upper) configuration. In Figure 7, the sudden stress drop is considered to be due to the aluminum fracture or interface separation. The sudden stress drop exhibited a similar trend in a previous study [36], where Kim et al. used copper/aluminum/copper cladding materials and performed three-point bending tests. The bending test confirmed that the stress drop section occurred because of the interface separation and fracture of copper and aluminum. These phenomena are discussed in detail later by the OM images. In the bending test of the DP590–A356 multi-material, a sudden stress drop was observed in the aluminum (upper) configuration, except for the case of an adhesion strength of 30 MPa. In the steel (upper) configuration, a stress drop was observed in all adhesion strength cases and a stress drop occurred twice at 10 MPa. Figure 7c,d show the bending test results of the SS330–A5052 multi-material. A sudden stress drop was observed in the steel (upper) configuration at an adhesion strength of 10 MPa and in all aluminum (upper) cases. As shown in Figure 7c,d, at 10 MPa, the stress drop section appeared twice. The first stress drop section occurred as the adhesive fractured, while the second section occurred as the interface separated.

The results of the bending test at the same stacking configuration showed that the flexural stresses of both multi-materials increased with the adhesion strength until the interface separation because the uniform deformation of the multi-materials was maintained according to the adhesion strength increase. In terms of stress drop, both multi-materials exhibited opposite bending properties according to the stacking. For the DP590–A356 multi-material in the steel (lower) configuration (i.e., steel under tension), the tendency of a sudden drop was decreased owing to the interfacial separation and aluminum fracture. In contrast, for the SS330–A5052 multi-material in the configuration of aluminum under tension, the number of sudden stress drops decreased because of the low ductility of A356, which was under tension and fractured without resisting the load. A5052, which is more ductile than A356, was not fractured owing to its high fracture resistance. These results can be explained by the occurrence of necking. A356 was broken without necking. However, A5052 extended to fracture after necking, which is similar to the bending result shown in Figure 6. 

### 3.3. Observation of the Fracture Behavior

**Figure 8:**
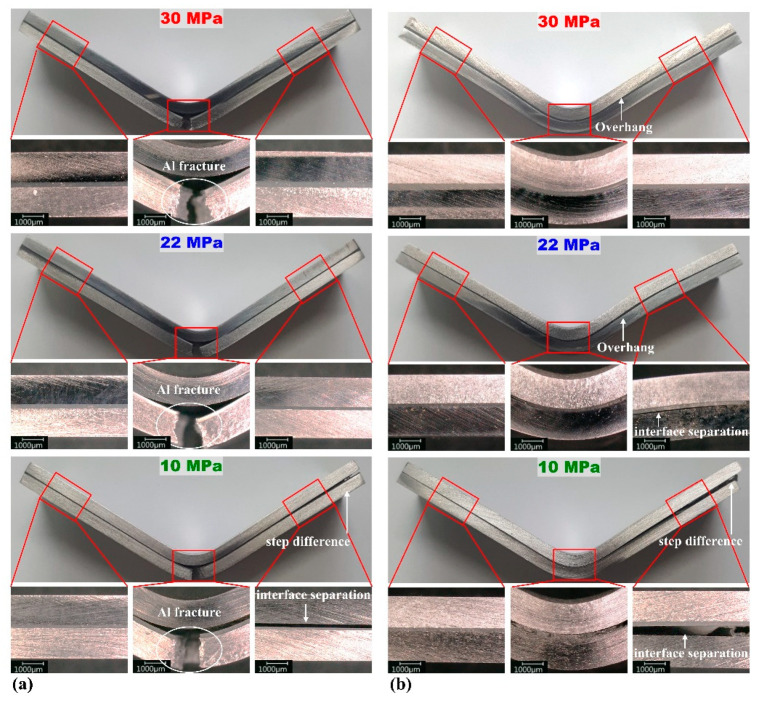
DP590–A356 multi-material after the flexure test: (**a**) steel (upper)–aluminum (lower) and (**b**) steel (lower)–aluminum (upper). The white circle indicates the Al fracture, while the white arrow indicates interface separation or step difference.

**Figure 9:**
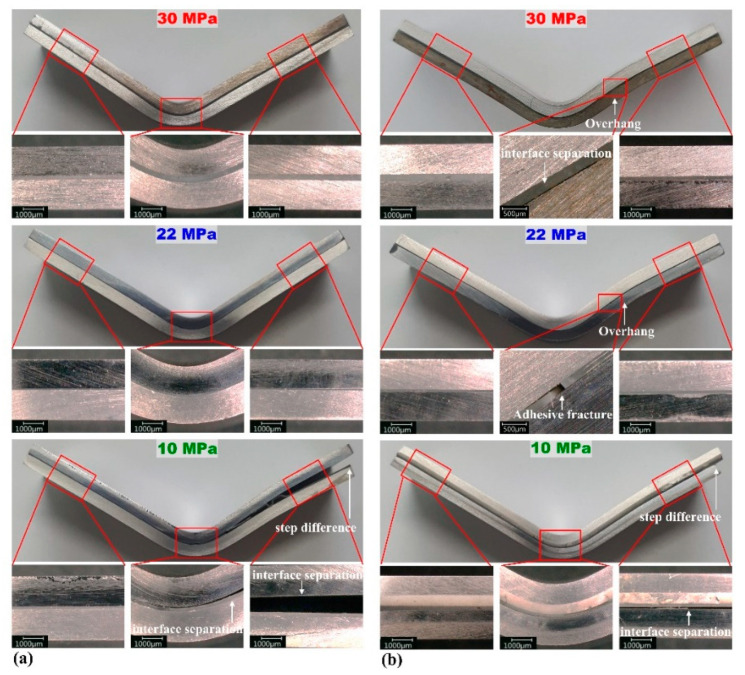
SS330–A5052 multi-material after the flexure test: (**a**) steel (upper)–aluminum (lower) and (**b**) steel (lower)–aluminum (upper). The white arrow indicates adhesive fracture, interface separation, or step difference.

Figure 8 and Figure 9 show the bending specimens of the multi-materials after the flexure test. The sudden stress drops in the flexure stress–displacement curves were related to the aluminum fracture (indicated by the white circle in Figure 8a), adhesive fracture, interface separation, or step difference (indicated by white arrows in Figure 8 and Figure 9). In Figure 8a, compared to Figure 7a, the stress drop section was caused by the aluminum fracture. Figure 8a (adhesion strength of 10 MPa) shows the aluminum fracture and interfacial separation, consistent with the result of the double stress drop section in Figure 7a at 10 MPa. At 20 and 10 MPa in Figure 7b, the stress drop section occurred by interface separation, as in Figure 8b. The same result is observed in Figure 7c and Figure 9a, which confirms that the interfacial separation occurred only at 10 MPa. Figure 7d demonstrates that the stress drop section appeared under all conditions, which is consistent with the result of the interface separation shown in Figure 9b. As the interface separation, the steel and aluminum deformed separately, which resulted in the step difference.

Under the conditions where a sudden stress drop was observed, bending was observed only on one overhang sheet (Figure 8 and Figure 9), owing to the bending moment, affected by the shear strength acting on the adhesive–metal interface. The bending of only one overhang sheet was reduced by increasing the adhesion strength and forming speed [37]. This bending behavior was consistent with the OM images; the sudden stress drop decreased as the adhesion strength increased. In addition, all overhangs were observed when the steel was under tension (lower). This is attributed to the difference in bending stress. As the stress applied to the specimen increased, overhangs were observed. Thus, if the steel is in the lower position, a larger stress is required for the same deformation.

**Figure 10:**
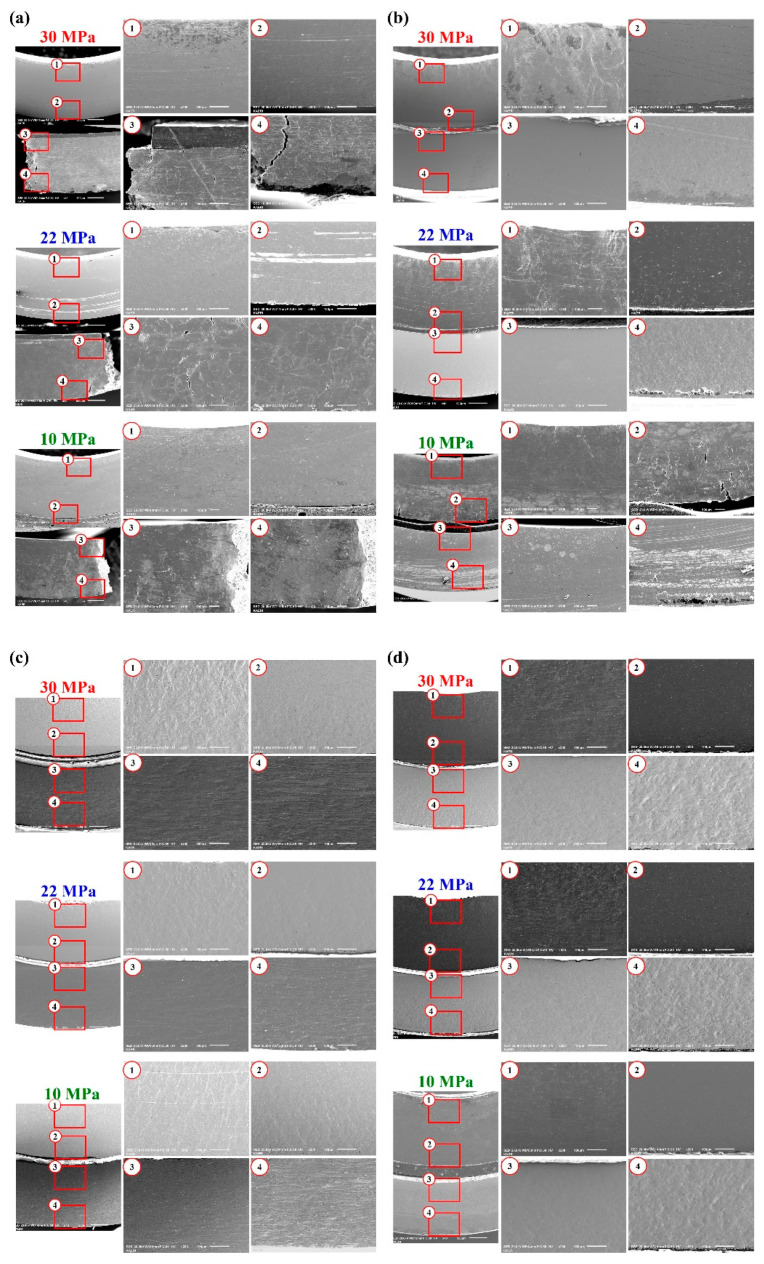
SEM images of the central areas in the multi-materials after the three-point bending test: (**a**) DP590 (upper)–A356 (lower), (**b**) DP590 (lower)–A356 (upper), (**c**) SS330 (upper)–A5052 (lower), and (**d**) SS330 (lower)–A5052 (upper).

Figure 10 shows SEM images of the central areas in the multi-materials after the three-point bending test. Figure 10a shows the DP590 (upper)–A356 (lower) configuration. As shown in Figure 10a, A356 fractured under all adhesion strengths. Adhesion peeling, which means interface separation, appeared only under an adhesion strength of 10 MPa. After the A356 fractured, DP590 was deformed in a single part and tension deformation was observed at site No. 2. Figure 10b shows the DP590 (lower)–A356 (upper) configuration. Under adhesion strengths of 22 and 30 MPa, site No. 1 showed that the deformation layer was under a compressive stress. Nos. 2 and 3 had relatively small deformations owing to the neutral layer around the adhesive, while No. 4 exhibited a large tensile deformation. There was a stress drop section at 22 MPa, as shown in Figure 7b, but there was no adhesive peeling or fracture in the SEM image, because adhesive fracture occurred near the supporting pin before the end of the test. However, at 10 MPa, A356 near the adhesive (site No. 2) exhibited a tensile deformation, which could be attributed to the relatively quick adhesive peeling. As the adhesive peeled off, the steel and aluminum deformed separately.

Figure 10c shows the SS330 (upper)–A5052 (lower) configuration. Adhesion peeling occurred under an adhesion strength of 10 MPa and the compressive stress led to the deformation layer at No. 1. SS330 exhibiting a tensile deformation at site No. 2, likely owing to the relatively rapid adhesive peeling. At 22 and 30 MPa, No. 1 exhibited a compressive deformation. However, Nos. 2 and 3 near the adhesive layer exhibited a lower deformation than those of Nos. 1 and 4. This indicates that the neutral layer was in the middle between Nos. 2 and 3. A deformation due to the large tension was observed at No. 4. Figure 10d shows the SS330 (lower)–A5052 (upper) configuration, where adhesive peeling was observed at 10 MPa. At 22 and 30 MPa, a stress drop zone occurred. However, it was not visible in the SEM image because adhesive peeling occurred at the edge rather than at the center of the specimen. Moreover, owing to the formation of a neutral layer between Nos. 2 and 3, the deformation was small compared to those at other sites. A compressive deformation was observed at No. 1, while a tensile deformation was observed at No. 4.

**Figure 11:**
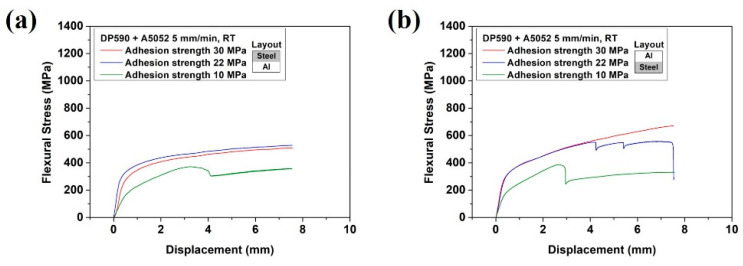
DP590–A5052 multi-materials with different adhesion strengths: (**a**) steel (upper)–aluminum (lower) and (**b**) steel (lower)–aluminum (upper).

The results are in accordance with those of the stacking structure configurations of both multi-materials. However, the opposite results were observed for DP590–A356 and SS330–A5052. To determine whether this tendency was attributed to the difference in strength between steel and aluminum or the ductility of aluminum, a bending test was performed by joining DP590 and A5052. Figure 11 shows the bending test results of the DP590–A5052 multi-materials with different adhesion strengths. For the steel (upper)–aluminum (lower) configuration, the tendency of the sudden stress drop decreased. This tendency was the same as that of the bending test on SS330–A5052 in Figure 7c,d. In addition, the bending strength improved as the strength of the lower material increased. Figure 11b (steel (lower)–aluminum (upper)) shows a higher flexure stress than that of the steel (upper)–aluminum (lower) configuration. These results are similar to those in the literature. When the soft material was set outside, the total thickness decreased with the increase in relative curvature and the bending moment was relatively low [38]. Based on these results, the stacking of the multi-material was determined by the ductility of aluminum rather than by the difference in strength between steel and aluminum. The bending characteristics had a larger influence on the mechanical properties of each single material and adhesion strength. Thus, the flexural stress was larger when a soft material was placed on the upper surface. For that reason, under the aluminum (upper)-steel (lower) condition, steel with a relatively high strength and elongation is located to the tensile load. Moreover, aluminum is located to a higher compressive load to reduce the occurrence of fracture. The adhesion strength was sufficient to prevent interface separation of the adhesive. However, if the adhesion strength was low, aluminum with a good ductility in the lower position exhibited better properties in terms of the stress drop tendency. Regarding the joining of steel and aluminum, not only the ductility of aluminum but also the strength of the adhesive must be considered.

**Figure 12:**
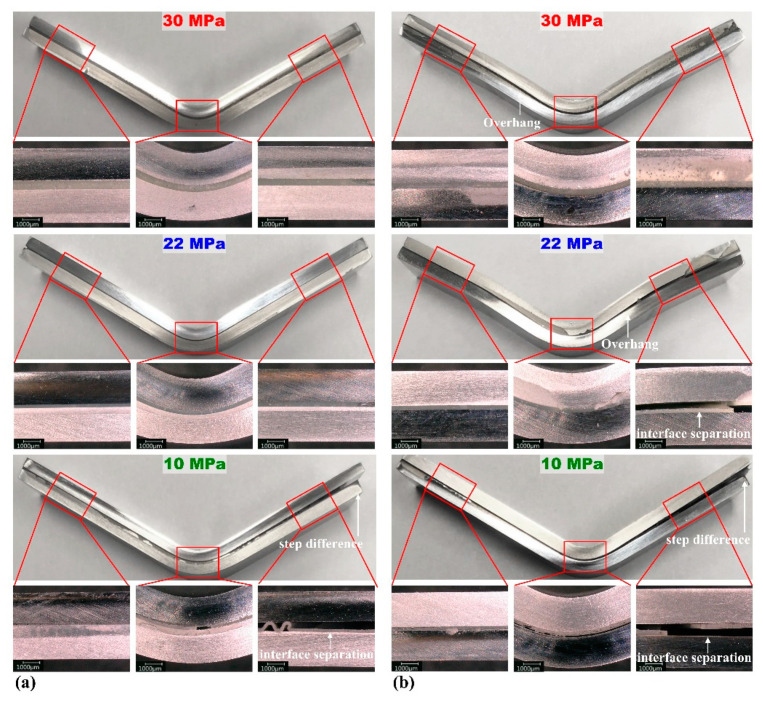
DP590–A5052 multi-material after the flexure test: (**a**) steel (upper)–aluminum (lower) and (**b**) steel (lower)–aluminum (upper). The white arrow indicates interface separation or step difference.

Figure 12 shows OM images of the specimens after the bending tests on DP590–A5052. The sharp stress drops in the flexure stress–displacement curves were related to interface fracture or separation, as reflected by the previous results. Moreover, interface separation can be observed in Figure 12a at an adhesion strength of 10 MPa and Figure 12b at adhesion strengths of 10 and 22 MPa. Figure 11b shows that, at 22 MPa, a sharp stress drop period appeared three times, but the OM image showed only one interface separation. This could be attributed to the interface separation after double fracture of the adhesive.

### 3.4. FEA Results

**Figure 13:**
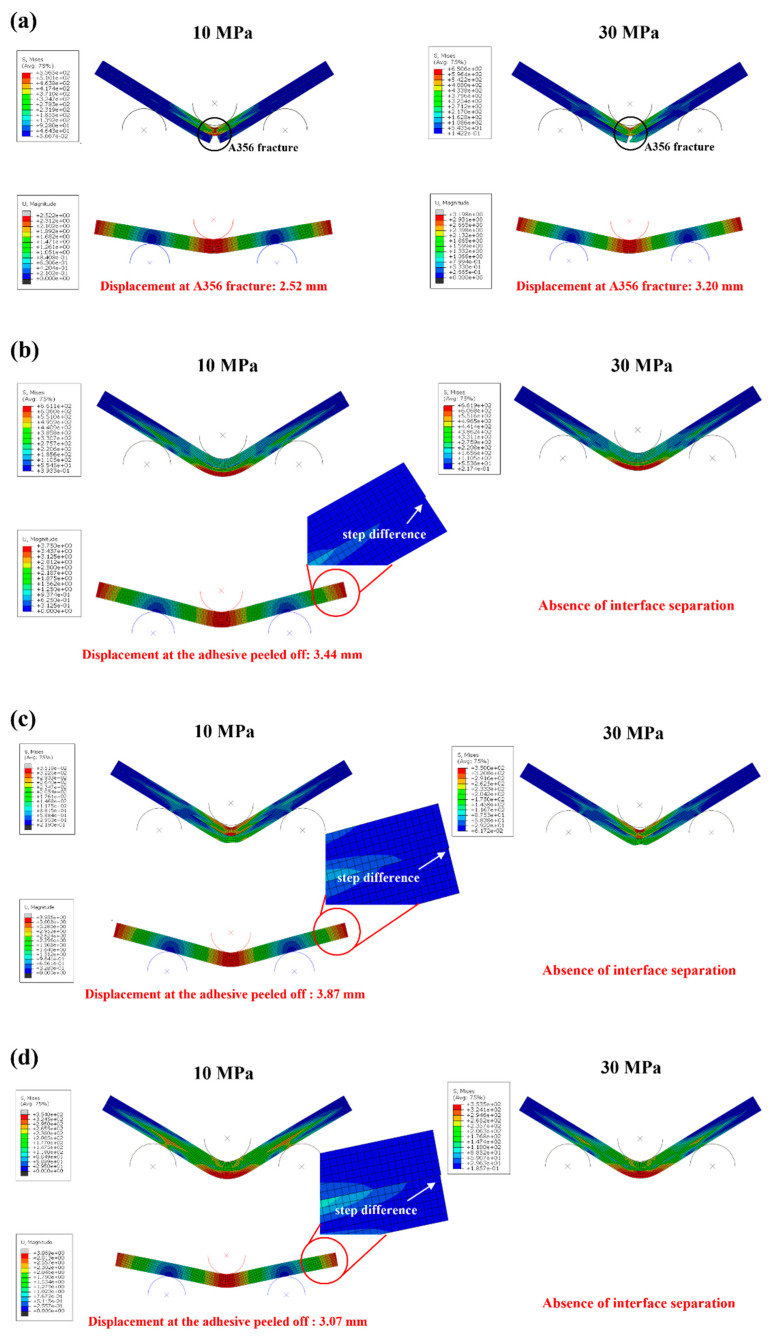
FEA results: (**a**) DP590 (upper)–A356 (lower), (**b**) DP590 (lower)–A356 (upper), (**c**) SS330 (upper)–A5052 (lower), and (**d**) SS330 (lower)–A5052 (upper).

The FEA was performed for comparison to the bending test results. In the FEA, the distribution of stress, the separation between the steel and aluminum, and the fracture results of A356 were analyzed. Figure 13 presents the results of the von Mises stress and displacement for each condition. The FEA results of DP590 (upper)–A356 (lower) are shown in Figure 13a. The fracture of A356 occurred under all conditions. The fractured displacements of A356 at 10 and 30 MPa were 2.52 and 3.20 mm, respectively. These results are very similar to the results of the bending test, as shown in Figure 7a. In addition, the stress was concentrated in DP590 after the A356 fracture. The fracture displacement of A356 was very similar to that of the bending test. Figure 13b shows the results of DP590 (lower)–A356 (upper). At an adhesion strength of 10 MPa, the adhesive peeled off at a displacement of 3.44 mm. This is comparable to the result of the experiment, where the adhesive peeled off at a displacement of 3.76 mm (Figure 7b). The separation of steel and aluminum could be observed at both ends of the specimen. At all adhesion strengths, there was no significant difference in the maximum von Mises stress. However, the stress distribution was more spread to both ends of the specimen at 10 MPa than at 30 MPa.

The results of SS330 (upper)–A5052 (lower) are shown in Figure 13c. There was no interface separation until the end of the bending test at 30 MPa. At 10 MPa, interface separation occurred at a displacement of 3.87 mm, similar to the displacement of 4.12 mm in Figure 7c. In addition, a neutral layer was formed between the steel and aluminum, which was also observed in the SEM image in Figure 10c. Figure 13d shows the results of SS330 (lower)–A5052 (upper). At an adhesion strength of 10 MPa, the separation between SS330 and A5052 was 3.07 mm, slightly different from the displacement of 3.68 mm in the bending test. In addition, at 30 MPa, there was no separation of the steel and aluminum until the end of the bending experiment. There was no difference in the maximum von Mises stress at 10 and 30 MPa. However, at 10 MPa, the stress distribution was spread to both ends of the specimen. A neutral layer was formed between the steel and aluminum, similar to the result shown in Figure 10d.

Based on the FEA results and bending test, the flexure stress increased when the steel was placed in the lower position. In other words, a smaller deformation occurred when the steel was in the lower position, even if the same force was applied to the multi-material. In addition, there was no separation between the steel and aluminum when the adhesion strength was sufficiently high. Thus, the setting of aluminum in the lower position is of interest when aluminum is sufficiently ductile. However, if the adhesion strength is low, it is more advantageous to set steel in the lower position.

**Figure 14:**
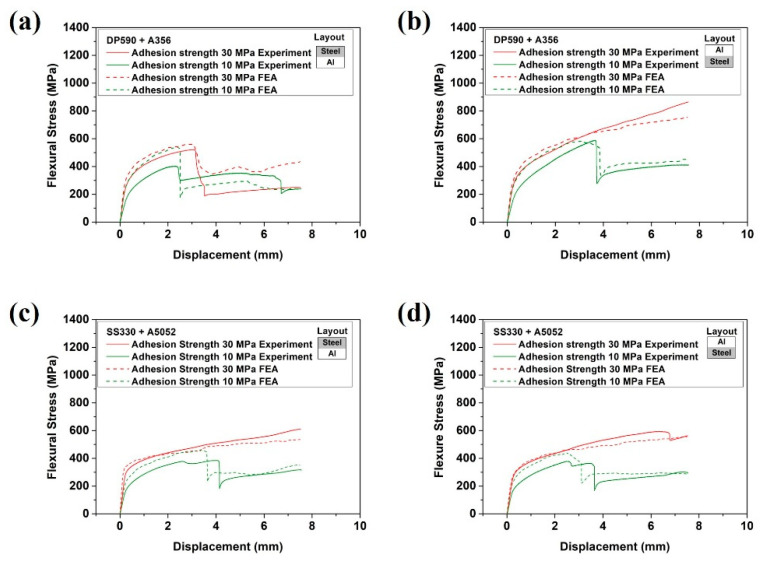
Flexural stress–displacement curves obtained by the FEA: (**a**) DP590 (upper)–A356 (lower), (**b**) DP590 (lower)–A356 (upper), (**c**) SS330 (upper)–A5052 (lower), and (**d**) SS330 (lower)–A5052 (upper).

To verify the FEA, the results of Figure 7 were compared to the flexure stress curves for each displacement obtained by the FEA, as shown in Figure 14. The results of the bending test are shown by the solid lines, while the data obtained by the FEA are shown by the dotted lines. All analysis data exhibited a similar trend to that of the experiment. The comparison of the bending and analysis results showed similar flexural stress values and stress drops in similar sections. The similarity of the bending stress values verified the material properties of FEA, and the similar stress drop sections verified the cohesive behavior parameters of FEA. In this manner, the validity of the interpretation was verified.

## 4. Conclusions

The multi-materials were obtained by joining steel and aluminum with a structural adhesive. Their bending properties were evaluated. The mechanical behaviors of the two multi-materials, DP590 (steel sheet)–A356 (cast aluminum alloy) and SS330 (steel sheet)–A5052 (aluminum sheet), were investigated. The following conclusions were obtained by the test results and FEA:

(1) The surface roughness, curing time, and temperature were increased to improve the adhesion strength from 22 to 30 MPa. In other words, to increase the adhesion strength, it was necessary to consider not only the surface roughness of the specimen, but also the curing time and temperature.
(2) According to the bending tests on the DP590–A356 and SS330–A5052 multi-materials, the bending stress increased with the adhesion strength. It was necessary to improve the adhesion strength for the joining of the two different materials. The stress drop section was more significant in DP590 (upper)–A356 (lower), SS330 (lower)–A5052 (upper), and DP590 (lower)–A5052 (upper). According to these results, the stress drop section was attributed to the ductility of aluminum, not to the difference in strength between steel and aluminum.
(3) According to the bending tests, if the adhesive was toughed to not cause adhesive peeling, the flexural stress was larger when the aluminum was placed on the upper side. However, if the adhesion strength was low, the setting of aluminum with a good ductility in the lower position provided better properties in terms of the stress drop tendency.
(4) The FEA results confirmed that the stress distribution was more spread at 10 MPa than at 30 MPa to both ends of the specimen. The flexural stress increased when the steel was placed at the lower position. In addition, the flexure stress–displacement curves obtained by the FEA and measured bending results were very similar, which verified the validity of the FEA results.

In conclusion, the ductility of aluminum and stacking dependent on the strength of the adhesive should be considered together in the joining of steel and aluminum. Furthermore, for experiments on the relationship between the adhesives and the multi-materials, fracture behavior analysis and microscopic observation for the deformation are required. Also, it is necessary to study the strain analysis of steel and aluminum, which are the base materials of adhesive-bonded multi-materials, and the fatigue failure behavior of multi-materials; this will be dealt with in a follow-up study.

## Figures and Tables

**Table 1:** Chemical compositions of the (a) DP590 and SS330 steels and (b) A356 and A5052 aluminum alloys used in this study (wt%).

(a)									
	**Si**		**C**	**P**		**Mn**		**Fe**	
DP590	0.3		0.1	0.01		1		Rem	
SS330	0.02		0.04	0.02		0.9		Rem	
(b)									
	**Si**	**Mg**	**Fe**	**Zn**	**Cu**	**Ti**	**Mn**	**Cr**	**Al**
A356	7.0	0.4	0.15	0.1	0.2	0.2	0.1	-	Rem
A5052	0.11	2.51	0.38	-	0.03	0.01	0.05	0.16	Rem

**Table 2:** YS, UTS, and EL of each single material.

	YS (MPa)	UTS (MPa)	EL (%)
DP590	374	632	29.8
SS330	275	356	48.9
A356	131	240	16.8
A5052	176	252	21.8

**Table 3:** Surface roughness of steel and aluminum (the Ra values are shown in μm).

	Steel	Aluminum
Bastard cut file	0.93	1.27
SiC paper #2000	0.02	0.11

**Table 4:** Bending test results of each single material.

	Flexural Stress at Maximum Load (MPa)	Flexural Displacement at Maximum Load (mm)	Flexural Displacement at Fracture (mm)
DP590	1004	7.5	Not fractured
SS330	523	7.5	Not fractured
A356	386	2.8	4.3
A5052	377	7.5	Not fractured

## Data Availability

The data presented in this study are available upon request from the corresponding author.
